# Phenotypic Variation of the Invasive Plant *Ageratum conyzoides* and Analysis of Its Competitiveness With the Co‐Occurring Indigenous Species *Perilla frutescens*

**DOI:** 10.1002/ece3.71532

**Published:** 2025-06-10

**Authors:** Xubo Chen, Yafen Zhang, Jianchun Chen, Zhengrong Luo, Yuqin Tan, Mengting Yu, Yiran Wu, Chengyu Zhou, Zhixin Xu

**Affiliations:** ^1^ College of Ecology Lishui University Lishui China; ^2^ School of Tourism Services and Management Tourism College of Zhejiang Hangzhou China

**Keywords:** *Ageratum conyzoides* L., competitiveness, *Perilla frutescens* (L.) Britt., phenotypic characteristic, phenotypic variation

## Abstract

*Ageratum conyzoides* is a malignant invasive plant in China. In this study, we sought to clarify the phenotypic variation of this plant and its competitive interaction with the indigenous species *Perilla frutescens* . Therefore, we established single‐species planting groups for each species, with plant densities of one, two, four, or eight individuals. In addition, we set up mixed‐species planting groups comprising one, two, or four individuals of both species. The results revealed significant differences in phenotypic characteristics of *A. conyzoides* , such as the mean aboveground biomass (AB) in all planting, specific stem length (SSL), and specific leaf area (SLA) in single‐species planting. Under the mixed‐species planting, the height of *P. frutescens* was significantly lower than *A. conyzoides* . The initial leaf length, plant height, and planting pattern of *A. conyzoides* were found to have a significant influence on AB, whereas the initial plant height had a significant influence on growth, and the planting pattern had a significant influence on SLA. For *A. conyzoides* , the coefficient of variation (*CV*) values of SSL in the low‐density mixed‐species planting (HZ_2_), flower bud intensity (FBI) in high‐density mixed‐species planting (HZ_8_), and AB in all mixed‐species planting patterns were greater than 20.0%, thereby indicating that *A. conyzoides* has strong plasticity. This comparison of competitiveness indicated that the interspecific competition between *A. conyzoides* and *P. frutescens* was greater than the intraspecific competition between the respective species and that the competitive capacity of *A. conyzoides* was greater than that of *P. frutescens* , particularly under conditions of the medium‐density mixed‐species planting. Based on these findings, we conclude that *A. conyzoides* can adapt to intraspecific and interspecific competition via phenotypic characteristics variation and maintain a competitive advantage. In addition, we established that in the presence of sufficient resources, the competitiveness of *A. conyzoides* is strongest at medium plant densities.

## Graphical abstract

*A. conyzoides* can adapt to intraspecific and interspecific competition via phenotypic characteristics variation under different planting patterns and maintain a competitive advantage when its niche overlaps with that of *P. frutescens* . In addition, we established that in the presence of sufficient resources, the competitiveness of *A. conyzoides* is strongest at medium plant densities.

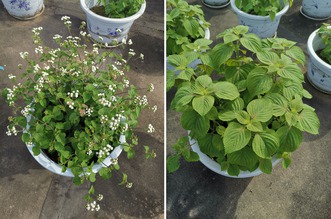

## 1 Introduction

Biological invasion has emerged as one of the major global environmental problems of the 21st century (Li et al. 2022; Negi et al. 2025). As an important invasive mechanism, phenotypic plasticity has become the core concept of ecological evolutionary and developmental biology studies and is also an important source of phenotypic variation (Wang and Zhou 2017; Khatri et al. 2023). Phenotypic plasticity is a strategy that facilitates the rapid adaptation of alien plants to new environments, which, in conjunction with genetic differentiation, can contribute to promoting the successful invasion of these exotic plants (Xiong and Zhao 2022). By altering their phenotypes, alien plants can acquire more resources from new environments to promote their growth, for example, by modifying their morphology, biomass allocation to different organs, and physiological characteristics (Zhang et al. 2017). However, given its complexity, the response of plant phenotypes to biological factors, such as density or competition, animal feeding, and microorganisms, is often overlooked in ecological research (Puy et al. 2021).

The successful invasion of alien plants is typically determined by the morphological, physiological, and life history characteristics of both invasive and local species (Gong et al. 2009; Negi et al. 2024; Khatri et al. 2024), the interactions among which can disrupt the ecological balance of the original local soil, thereby affecting the relative competitive capacities of these plants (Berger et al. 2008). Consequently, the relationships between alien plants, particularly invasive types, and native species have attracted considerable attention (Zhao et al. 2019), as studies on competitive relationships can provide a basis for ecological prevention and control (Wang et al. 2015; Chen et al. 2023), and indeed, some such studies have succeeded in quantifying the degree of competitiveness (Wang 2021; Xu and Ma 2021; Pan et al. 2022).

To date, however, research has tended to focus primarily on utilizing changes in biomass to measure competitive relationships, whereas there has been relatively little research on the plastic changes in the phenotypic characteristics of invasive species associated with changes in competitive relationships, such as plant height, specific leaf area (SLA), bud strength, and specific stem length (SSL). The plasticity of these phenotypic characteristics plays key roles in the invasion process and is directly related to species coexistence and the maintenance of biodiversity (Wang et al. 2012; Valladares et al. 2015). Consequently, to further elucidate the invasive capacities of alien species, it is necessary to study the plastic changes or phenotypic variations associated with the competition between invasive and native plants.

*Ageratum conyzoides* L., a herbaceous plant in the family Asteraceae native to Central America and Mexico, has invaded natural, urban, and agricultural ecosystems in numerous countries throughout tropical and subtropical regions worldwide (Negi et al. 2020; Chen et al. 2025). As an invasive alien, it interferes with crops, grasslands, and forest flora, and its capacity to displace native plant species is a source of considerable concern (Kaur et al. 2023). In South China, for example, *A. conyzoides* has caused serious ecological harm, and previous studies have found that this plant is characterized by strong phenotypic plasticity with respect to its adaptability to different abiotic factors, such as shade and soil nutrients (Xu et al. 2019). Moreover, its comparatively large niche width and the generally low competitive resistance from local plants have resulted in the extensive and severe invasion in Lishui, Zhejiang Province, China (Zhang et al. 2022). Previously, we have experimentally examined the competitive relationship between *A. conyzoides* and *Perilla frutescens* (L.) Britt. (1:4 mixture) and found that the growth and reproduction of *A. conyzoides* were generally inhibited by *P. frutescens* when grown at small competitive distances, with lower phenotypic plasticity. However, with an increase in competitive distance, *A. conyzoides* rapidly adapted to the interspecific competition and was characterized by higher phenotypic plasticity (Chen et al. 2023). Given that the ecological niche overlap between *A. conyzoides* and native weeds is significantly higher than that among native weeds (Zhang et al. 2022), assessing the interspecific competition between *A. conyzoides* and native species under different planting patterns and the associated phenotypic variation can contribute to gaining a better understanding of the invasive mechanisms of *A. conyzoides* , which would be of considerable practical value. During field investigations, we have observed that *A. conyzoides* frequently co‐occurs with *P. frutescens* , and, consequently, we assume that when facing competition from *P. frutescens* , the phenotypic characteristics of *A. conyzoides* undergo certain changes to promote competitiveness. Accordingly, in this study, we sought to investigate the phenotypic variation and competitive performance of *A. conyzoides* in response to the presence of *P. frutescens* based on assessments under different planting patterns. Our aim was to elucidate how *A. conyzoides* generates adaptive phenotypic variations in the context of interspecific competition and thereby facilitates invasion. Such insights will contribute to gaining a more comprehensive understanding of the invasive mechanisms of *A. conyzoides* and, in doing so, provide a basis for prevention and control management.

## 2 Materials and Methods

### 2.1 Experimental Materials

**FIGURE 1:**
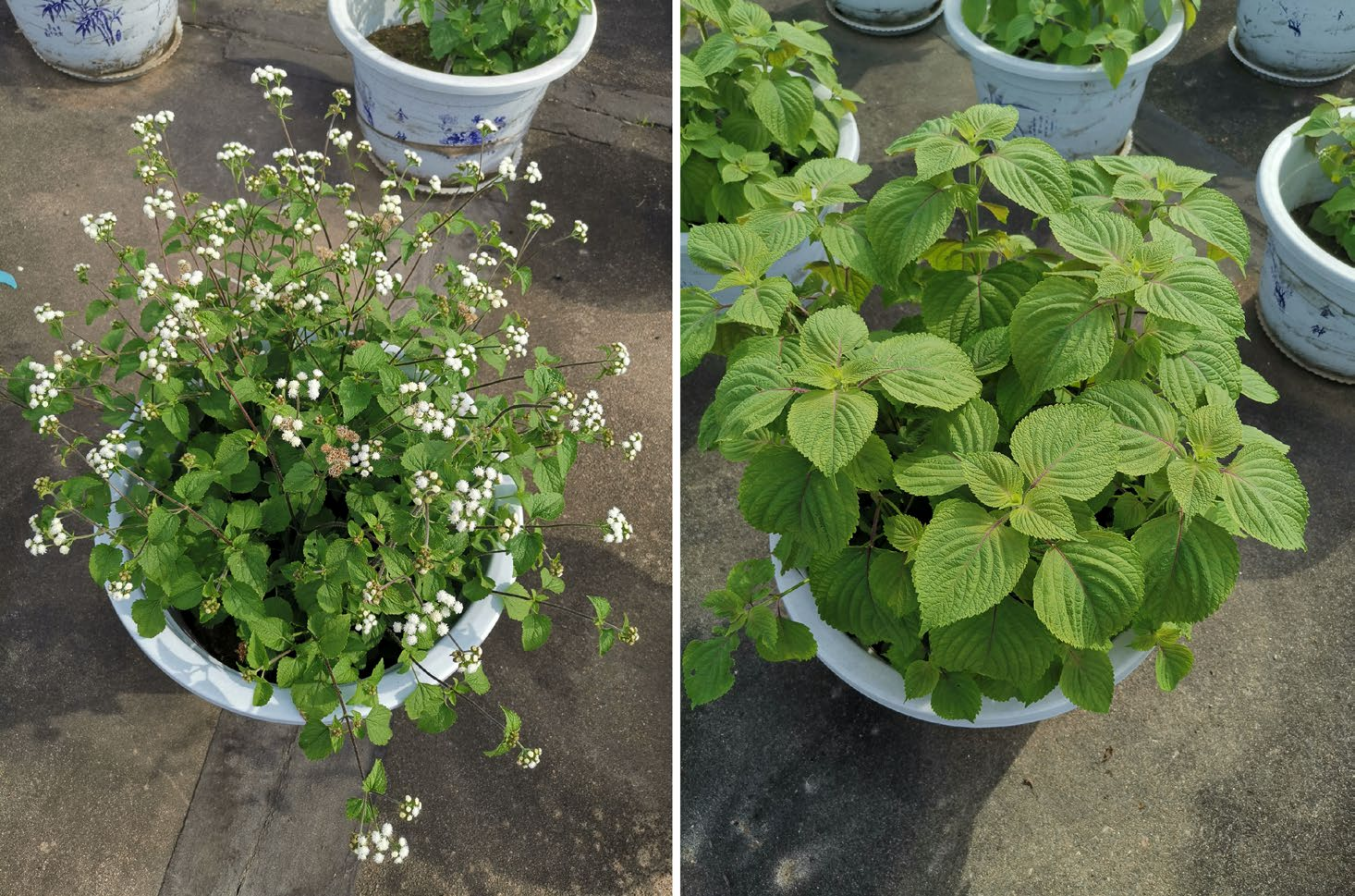
The morphological characteristics of the two species.

Seeds of *A. conyzoides* and *P. frutescens* (from the same population) were harvested from the field in Liandu District, Lishui City, Zhejiang Province, China, and were subsequently sown in pots, with the seedlings being cultivated under natural conditions on the rooftop of the Lishui University laboratory building. The morphological characteristics of the two plant species are presented in Figure 1, with *A. conyzoides* displayed in the left panel and *P. frutescens* shown in the right panel.

### 2.2 Experimental Methods

#### 2.2.1 Experimental Design

Plants were transplanted on May 3, 2022, and the experiment period ran from May 3 to July 20. Seedlings of similar heights and leaf sizes were carefully selected and transplanted to soil‐containing plastic pots (inner diameter, 45 cm; bottom diameter, 29 cm; height, 34 cm). Each pot contained equal amounts of soil, which comprised a mixture of field soil, perlite, and peat, with a pH of 4.46, and contents of slow‐release potassium, fast potassium, total nitrogen, hydrolyzed nitrogen, water content, organic matter, and available phosphorus of 580 mg/kg, 140 mg/kg, 0.4%, 210 mg/kg, 2.5%, 85.7 g/kg, and 155 mg/kg, respectively. The experimental design was based on single‐species planting treatments for *A. conyzoides* and *P. frutescens* , each comprising treatment groups of one, two, four, and eight individuals (designated at H_1_, H_2_, H_4_, and H_8_ for *A. conyzoides* and Z_1_, Z_2_, Z_4_, and Z_8_ for *P. frutescens* , respectively). In addition, we assessed the mixed‐species planting of *A. conyzoides* and *P. frutescens*, with one, two, and four individuals of each species in a 1:1 ratio, designated HZ_2_, HZ_4_, and HZ_8_, respectively. For each of the 11 treatment groups, we set up four replicates. With the center of the pot as the center and a radius of 11 cm, the plants were evenly distributed on the circumference. With respect to the mixed‐species groups, we mainly focused on simultaneously increasing the number of competing and native species in a 1:1 ratio to observe their phenotypic variations and changes in competitiveness. Plant pots were placed randomly, and the spacing between the plant pots, which were placed on the roof of the aforementioned experimental building, was maintained at approximately 50 cm. Two days after planting, measurements were obtained for initial plant height and leaf length. During the period of cultivation, the plants in each treatment were irrigated with sufficient amounts of water to ensure that the soil was moist and timely rid of diseases, pests, and weeds.

#### 2.2.2 Determination of Phenotypic Characteristics

For the purposes of this study, we assessed the following phenotypic characteristics. The initial length of leaves and increases in plant height were measured using a ruler (accurate to 0.1 cm). For other characteristics, plants were cut from the base and brought back to the laboratory. For the measurement of leaf area (excluding petioles), three mature leaves from each *A. conyzoides* plant were obtained from the branch below the top inflorescence (i.e., the uppermost branch) and three mature leaves from *P. frutescens* plants were collected from the top of the main stem. Copies of these leaves, obtained using a photocopier, were then used to determine the leaf area using a CI‐203 laser leaf area instrument. In addition, branches were detached from the main stems of plants, weighed individually, and measured (with an accuracy of 0.1 cm), and the numbers of *A. conyzoides* buds were counted. Having obtained these measurements, the main stems, branches, and leaves used for leaf area determinations were respectively placed in a blast dryer and dried at 80°C to a constant weight, with an electronic balance (accurate to 0.001 g) being used to measure the dry weights of the leaves, branches, and main stems of each plant.

#### 2.2.3 Calculation of Different Indices

Increases in the relative heights of plants were determined as the natural logarithm of the height of the plant at harvest minus the natural logarithm of the height at planting. The mean aboveground biomass (AB) per plant was the sum of the average branch biomass, main branch biomass, and leaf biomass (as measured by leaf area). The SSL is the ratio of the main branch length (cm) to the main branch biomass (g) (Yu et al. 2020; Chen et al. 2023), the SLA is the ratio of leaf area (cm^2^) to leaf biomass (g) (Chen et al. 2023), and bud intensity is the ratio of the number of buds to the AB (g) (Chen et al. 2023). The competitive relationship between species was assessed in terms of the relative yield (*RY*) and the attack coefficient of competition (*A*) (Weigelt and Jolliffe 2003; Lv et al. 2011; Pan et al. 2022). *Y*_HZ_ is the average biomass per plant of *A. conyzoides* when grown with *P. frutescens* , *Y*_H_ is the average biomass per plant of *A. conyzoides* in single‐species planting, *Y*_ZH_ is the average biomass per plant of *P. frutescens* when grown with *A. conyzoides*, and *Y*z is the average biomass per plant of *P. frutescens* in single‐species planting. The formulae used to determine *RY* and *A* are as follows:

$${\mathrm{RY}}_{\mathrm{HZ}}=\frac{Y_{\mathrm{HZ}}}{Y_{H}}$$

$${\mathrm{RY}}_{\mathrm{ZH}}=\frac{Y_{\mathrm{ZH}}}{Y_{Z}}$$

$$A={\mathrm{RY}}_{\mathrm{HZ}}-{\mathrm{RY}}_{\mathrm{ZH}}$$

### 2.3 Data Processing and Statistical Analysis

One‐way analysis of variance combined with the least significant difference method (LSD) was used to analyze the differences in each index under different planting patterns using SPSS 18.0 statistical software. *t*‐tests were used to compare the differences between single‐ and mixed‐species plantings. The effects of initial leaf length, initial plant height, and planting pattern on phenotypic characteristics were assessed using multivariate regression analysis.

## 3 Results and Analysis

### 3.1 Variations in Plant Phenotypic Characteristics Under Different Planting Patterns

**FIGURE 2:**
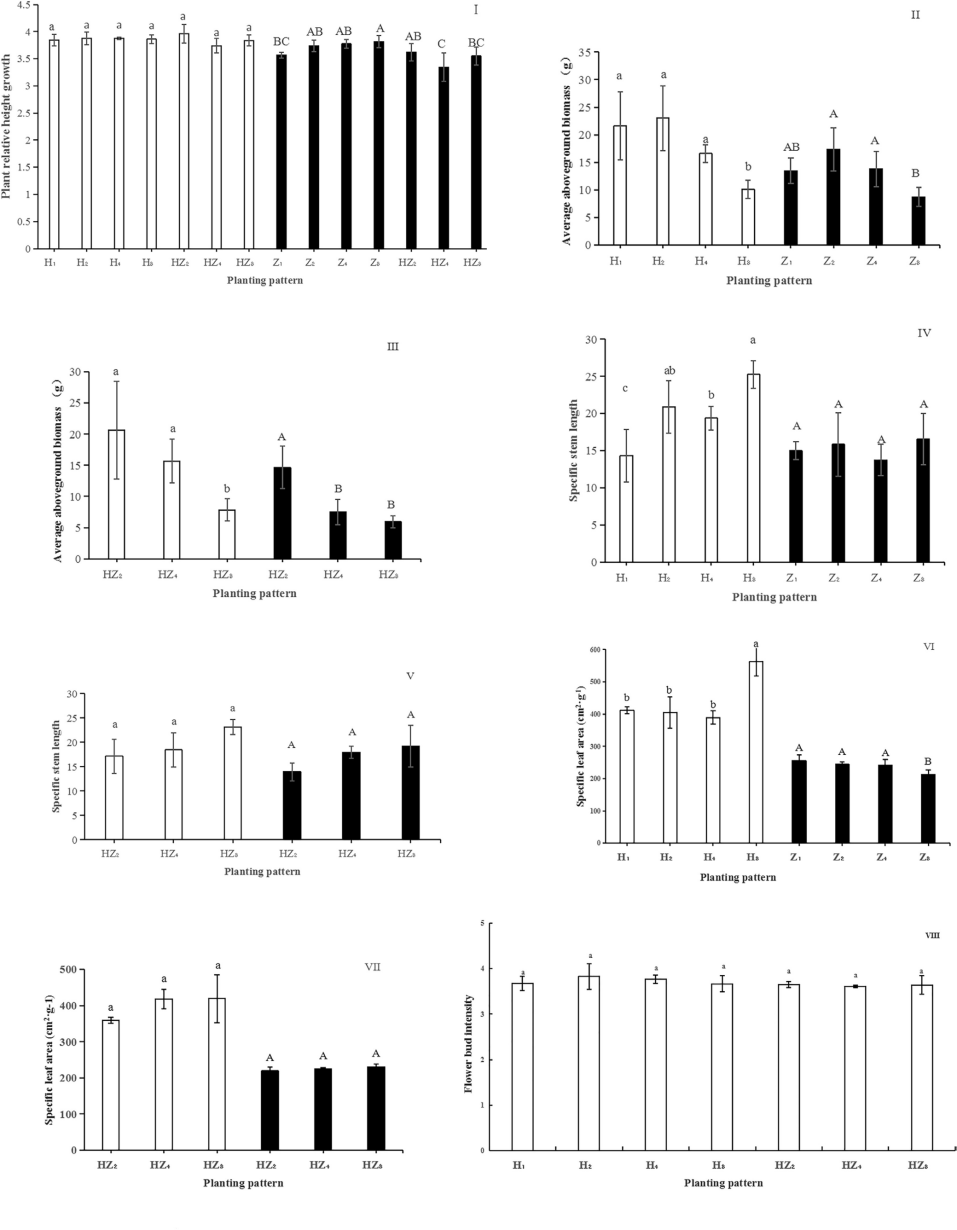
Variations in phenotypic characteristics of *Ageratum conyzoides* and *Perilla frutescens* under different planting patterns. The length of the bars in the histogram represents standard error. The same letters above the error bars indicate that there are no significant differences between planting patterns, and different letters indicate significant differences at the *p* < 0.05 level. Lowercase letters indicate the differences in the phenotypic characteristics of *A. conyzoides* , and uppercase letters indicate differences in the phenotypic characteristics of *P. frutescens* among different planting patterns. □ *A. conyzoides* ■*P. frutescens*.

When planted at different densities, we detected no significant differences with respect to increases in the relative heights of *A. conyzoides* , whereas there was a notable variation in the relative heights of *P. frutescens* grown under different planting patterns (Figure 2I). However, during the course of the experimental period, the growth in height of *A. conyzoides* plants was found to be significantly greater than that of *P. frutescens* (*p* < 0.05). As shown in Figure 2II,III, there were significant differences in the mean biomass of the aboveground parts of *A. conyzoides* or *P. frutescens* when grown at different planting patterns (*p* < 0.05), with significant reductions in the mean values of AB being observed with an increase in plant density. Under high‐density single‐ and mixed‐species patterns, compared with the maximum value, there were reductions of 56.3% and 62.0%, respectively, in the mean biomass of the aboveground parts of *A. conyzoides* and corresponding reductions of 49.7% and 59.4% for *P. frutescens*. Biomass comparisons revealed that the mean biomass of *P. frutescens* was significantly lower under medium‐density mixed‐species planting (HZ_4_) than under medium‐density single‐species planting (Z_4_), whereas there were no significant differences between single‐ and mixed‐species plantings under the same density treatments in *A. conyzoides* , thereby indicating that the HZ_4_ planting pattern had a considerable effect on the mean biomass of *P. frutescens* (Figure 2II,III).

As shown in Figure 2IV,V, there were significant differences in the SSL of *A. conyzoides* in single‐species planting treatments (*p* < 0.05), and the SSL under single‐plant planting was significantly lower than that under multiple‐plant patterns, however, there was no significant difference among the different mixed‐species planting patterns. *t*‐test analyses revealed that there were no significant differences in SSL between low‐density single‐species and mixed‐species plantings (H_2_ and HZ_2_), medium‐density single‐species and mixed‐species plantings (H_4_ and HZ_4_), or high‐density single‐species and mixed‐species plantings (H_8_ and HZ_8_). These findings accordingly indicated that the differences in the SSL of *A. conyzoides* were unrelated to whether the neighboring plants were conspecific or interspecific. However, under mixed‐species planting patterns, growth in the height of *P. frutescens* was significantly lower than that of *A. conyzoides* , thereby tending to indicate that *P. frutescens* showed no obvious compensatory response to shading by *A. conyzoides* .

As shown in Figure 2VI,VII, there were significant increases in the SLA of *A. conyzoides* when grown under the high‐density single‐species planting pattern, whereas differences were not significant under the different mixed‐species planting patterns. This difference can be attributed to the rapid growth of *A. conyzoides* , and the fact that the height of *A. conyzoides* was significantly higher than that of *P. frutescens* (*p* < 0.01). This advantage accordingly enables *A. conyzoides* to obtain sufficient light resources without the necessity of increasing its SLA when grown under conditions of mixed‐species planting patterns. However, under the high‐density single‐species planting pattern, we detected a significant reduction in the SLA of *P. frutescens* ; differences were non‐significant under different mixed‐species planting patterns, thereby providing evidence to indicate the minimal variability in this phenotypic characteristic.

To assess the changes in flower bud intensity (FBI) under different planting patterns, we used the natural logarithm of FBI, which revealed an absence of any significant differences in bud intensity among the different planting patterns (Figure 2VIII). The analyses revealed that when the mean value of AB changes significantly, the breeding input can be guaranteed preferentially.

### 3.2 Effects of Initial Factors and Planting Patterns on the Phenotypic Characteristics of *A. conyzoides*

Multivariate analysis of the effects of initial leaf length, initial plant height, and planting patterns (single‐ and mixed‐species planting) on relative height, mean AB, SSL, SLA, and bud intensity revealed that planting patterns and initial leaf length had significant effects on mean AB, where the initial plant height had a significant influence on the mean AB per plant and the relative height growth, and planting patterns had a significant influence on SSL (Table 1).

**TABLE 1:** Multivariate regression analysis of the initial factors and phenotypic characteristics of *Ageratum conyzoides*.

Phenotypic characteristics	Initial leaf length	Initial plant height	Planting pattern
Plant relative height growth (PHG)	0	**	0
Mean aboveground biomass per plant (AB)	**	*	**
Specific stem length (SSL)	0	0	*
Specific leaf area (SLA)	0	0	0
Flower bud intensity (FBI)	0	0	0

** Extremely significant effect (*p* < 0.01).

* Significant effect (*p* < 0.05); 0 non‐significant effect.

### 3.3 Coefficient of Variation in Phenotypic Characteristics of *A. conyzoides* and Its Competition With *P. frutescens* Under Mixed‐Species Planting Patterns

**FIGURE 3:**
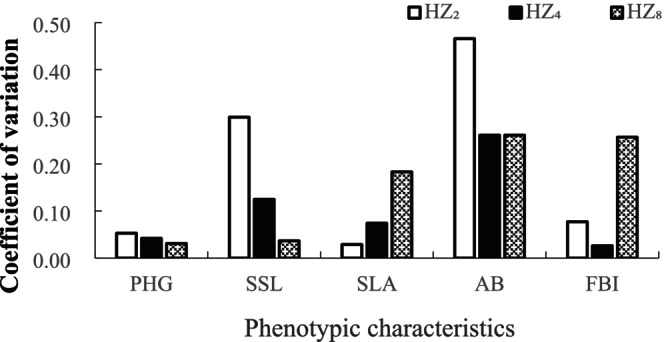
Coefficient of variation of phenotypic characteristics of *Ageratum conyzoides* in different planting patterns.

In addition to a relatively low variation coefficient for the growth of *A. conyzoides* , we also recorded high variation coefficients for the different phenotypic characteristics of this species in the different mixed‐species planting patterns, among which the *CV* values of SSL in the low‐density (HZ_2_) pattern, bud strength in the high‐density (HZ_8_) pattern, and the AB under all mixed‐species planting patterns were greater than 20.0%, with values ranging from 26.0% to 47.0%, indicating good plasticity (Figure 3). Moreover, the *CV* values obtained for SLA and bud strength under the HZ_8_ pattern were found to be significantly higher than those under the other mixed‐species planting patterns (*p* < 0.01). These findings thus indicate a considerable variation in the SLA and FBI of *A. conyzoides* in response to the influence of competition.

As shown in Table 2, when *A. conyzoides* and *P. frutescens* were grown under the three mixed‐species planting patterns, the *RY*_HZ_ and *RY*_ZH_ values were all less than 1, with the latter index being significantly less than 1 under the medium‐density mixed‐species planting pattern (HZ_4_) (*p* < 0.05). This accordingly indicates that the competitiveness of *A. conyzoides* and *P. frutescens* was less pronounced when grown under single‐species planting conditions (i.e., interspecific competition was greater than intraspecific competition). Moreover, we found that the value of the attack coefficient of *A. conyzoides* against *P. frutescens* was greater than 0, and significantly greater than 0 under the HZ_4_ planting pattern (*p* < 0.01), thereby providing evidence that under these conditions, *A. conyzoides* was more competitive than *P. frutescens* .

**TABLE 2:** Comparison of the competitiveness between *Ageratum conyzoides* and *Perilla frutescens* under different mixed‐species planting patterns.

Mixed‐species planting pattern	Relative yield of *A. conyzoides* (*RY*_HZ_)	Relative yield of *P. frutescens* (*RY*_ZH_)	Attack coefficient of competition (*A*)
HZ_2_	0.896	0.846	0.050
HZ_4_	0.948	0.546*	0.402**
HZ_8_	0.780	0.683	0.097

* Significant difference compared with 1.

** Very significant difference compared with 0.

## 4 Discussion

Invasive plants are frequently characterized by high seed productivity, small size, high rates of seed germination, survival, and growth, and a prolific reproductive capacity, which are all conducive to their superior utilization of resources for growth (Van Kleunen et al. 2010; Negi et al. 2023). Increases in the height of *A. conyzoides* plants are notably rapid under different planting patterns (as demonstrated in the present study) and spacing (Chen et al. 2023). This growth characteristic has a direct influence on the adaptability of its SLA under different planting patterns. SLA is associated with the utilization of light energy by plants, and the findings of previous studies have indicated that when exposed to heterologous or homologous neighbors in invaded areas, invasive plants can enhance their environmental adaptability by suitably modifying the plasticity of traits associated with resource utilization (Davidson et al. 2011; Yu et al. 2020). In the present study, we found that in response to an increase in planting density, there were corresponding increases in the intensity of competition for light resources and the SLA of *A. conyzoides* increased notably when grown under a single‐species planting pattern. Nevertheless, the growth rate of *A. conyzoides* was significantly higher than that of *P. frutescens* , and thus no significant differences were observed in the SLA of plants among mixed‐species planting patterns. Plant density can influence plant growth by affecting the total amounts of nutrient resources available to plants (Liu et al. 2022), and in this regard, it has been found that the growth of native plants in the presence of one alien plant individual was significantly better than that in the presence of two alien plant individuals (Ferenc et al. 2021).

Our findings in the present study revealed a significant reduction in the mean biomass of the aboveground parts of *A. conyzoides* when grown at a high density in the presence of a local species, which was similarly observed when *A. conyzoides* is exposed to intraspecific competition. However, this decline was more evident under conditions of interspecific competition. In the presence of conspecific competitors, *A. conyzoides* obtain more light resources by promoting a significant increase in its shade‐avoidance response (increasing SSL), whereas, contrastingly, we detected no significant difference in SSL under the different planting patterns when grown in the presence of *P. frutescens* , which can be attributed to the relatively higher growth rate of *A. conyzoides* . These findings tend to contrast with those obtained in our previous research, in which we observed the strongest shade avoidance response and a significant increase in SSL when a single plant of *A. conyzoides* was grown in the presence of four *P. frutescens* plants at a plant spacing of 5 cm compared with 2.5 cm (Chen et al. 2023), which could presumably be attributable to the inhibitory effects of the close proximity of multiple *P. frutescens* plants.

FBI serves as an indicator of flower bud production per unit of AB, and hence the proportion of reproductive input. The number of fruits and seeds produced by plants contributes to the reproductive fitness of invasive plants and thus determines the invasive capacity of these plants (Alpert et al. 2000). In the face of competition from conspecific plants or those of the native plant *P. frutescens* , *A. conyzoides* can prioritize reproductive input, which is a manifestation of its highly invasive ability. Our findings in this regard further verify that alien plants can adapt to new environments by adjusting phenotypic characteristics, such as plant height, biomass, and number of inflorescences, which is conducive to their colonization and spread (Rathee et al. 2021).

The initial status of an invasive plant can have either positive or negative effects on its phenotypic characteristics. The initial plant height, leaf length, and planting pattern of *A. conyzoides* can influence the acquisition of light resources, thereby potentially resulting in asymmetric competition for light and thus having significant effects on associated phenotypic characteristics. Asymmetric competition arising as a consequence of differences in initial height and initial leaf length can be understood in terms of size‐dependent asymmetric competition (Schwinning and Weiner 1998), which has a direct influence on the symmetry of the competitive relationship (Keddy et al. 2000) and is inextricably linked with the occurrence of invasion and species coexistence (Skácelová and Lepš 2014). This type of competition can be influenced by multiple factors, among which morphological plasticity is an important feature. In this regard, the coefficient of variation (*CV*) of a given phenotype is often used to determine the value of morphological plasticity (Valladares et al. 2006). With respect to *A. conyzoides* , we found that when grown in different mixed‐species patterns, the SSL, mean biomass of aboveground parts, and FBI of this invasive plant were characterized by a higher *CV*, which is assumed to reflect a better adaptability to competition from *P. frutescens* . As a consequence of the influence of competition, we observed a considerable variation in the SLA and FBI of *A. conyzoides* , thereby ensuring a requisite level value of reproductive fitness. In response to a reduction in the mean biomass of the aboveground parts, a higher rate of differentiation in FBI can serve as a strategy for adjusting reproductive fitness, and our findings in this study further confirmed that phenotypic variation in the flower traits of invasive plants reflects their environmental adaptability and thereby influences the reproductive fitness and invasive capacity of these plants (Alpert et al. 2000; Zhang and Lou 2022).

The competitive capacities of both invasive and native plants are primarily influenced by biomass allocation (Yang et al. 2024), and the intensity of interspecific competition is often determined based on AB (Puy et al. 2021). In the present study, we accordingly used the RY of the AB as an index to assess the competitive relationship between *A. conyzoides* and *P. frutescens* . Values obtained for the *CV* of AB were found to be larger in the mixed‐species planting groups, thereby indicating a substantial fluctuation in biomass as a consequence of the competition between these two plant species. Consistently, the values obtained for RY and competition coefficients revealed that interspecific competition was greater than intraspecific competition, and that *A. conyzoides* was more competitive than *P. frutescens* . Moreover, we detected clear differences in competitive relationships corresponding to differences in plant spacing. Under a low‐density mixed‐species planting pattern, the RYs of *A. conyzoides* and *P. frutescens* were both close to 1, and the intensity of competition between the two species was weak, which can be attributed to the relatively large distance (a linear distance of 22 cm) between plants of the two species and the availability of sufficient resources. Under the mixed‐species planting pattern at medium density, whereas the RY of *A. conyzoides* was again close to 1, that of *P. frutescens* was significantly less than 1, thereby revealing an increase in the intensity of competition between the two species. However, owing to a limitation in available resources, the growth of both species was restricted when planted at a high density. Although the observed reduction in the RY of *A. conyzoides* was less than that of *P. frutescens* , there was a notable reduction in the degree of its competitive advantage, which further supports our previously stated contention that external resource conditions play an important role in determining the invasive capacity of *A. conyzoides* (Luo et al. 2018).

## 5 Conclusions

Our findings in this study revealed considerable variability in the phenotypic characteristics of *A. conyzoides* under conditions of either intraspecific or interspecific competition, along with certain differences between intraspecific and interspecific competition. With respect to intraspecific competition, access to light resources can be enhanced by changes in SLA and stem length, whereas under conditions of interspecific competition, a combination of high plasticity in SLA, SSL, mean AB per plant, and FBI with a high growth ability was found to contribute to the maintenance of the competitive advantage of *A. conyzoides* when its ecological niche overlapped with that of the native species *P. frutescens* . Moreover, we established that an abundance of resources and moderate plant density were most conducive to the competitiveness and invasive capacity of *A. conyzoides* . Our characterization of the phenotypic plasticity and competitiveness of *A. conyzoides* will provide a valuable foundation for the future control of this alien species.

## Author Contributions

**Xubo Chen:** conceptualization (equal), data curation (lead), formal analysis (lead), funding acquisition (equal), investigation (equal), methodology (equal), project administration (lead). **Yafen Zhang:** data curation (equal), funding acquisition (equal), investigation (equal), methodology (equal), writing – original draft (equal). **Jianchun Chen:** data curation (supporting), investigation (equal), writing – original draft (supporting). **Zhengrong Luo:** conceptualization (equal), data curation (equal), formal analysis (equal), funding acquisition (equal), methodology (equal), writing – review and editing (equal). **Yuqin Tan:** data curation (supporting), investigation (equal). **Mengting Yu:** data curation (supporting), investigation (equal). **Yiran Wu:** data curation (supporting), investigation (equal). **Chengyu Zhou:** investigation (equal). **Zhixin Xu:** investigation (equal).

## Conflicts of Interest

The authors declare no conflicts of interest.

## Data Availability

The data that support the Figures 2 and 3 will be openly available in Dryad at https://doi.org/10.5061/dryad.931zcrjxt.

## References

[ece371532-bib-0001] Alpert, P. , E. Bone , and C. Holzapfel . 2000. “Invasiveness, Invasibility and the Role of Environmental Stress in the Spread of Non‐Native Plants.” Perspectives in Plant Ecology Evolution and Systematics 3, no. 1: 52–66.

[ece371532-bib-0002] Berger, U. , C. Piou , K. Schiffers , and V. Grimm . 2008. “Competition Among Plants: Concepts, Individual‐Based Modelling Approaches, and a Proposal for a Future Research Strategy.” Perspectives in Plant Ecology, Evolution and Systematics 9, no. 3/4: 121–135.

[ece371532-bib-0043] Chen, X. B. , J. C. Chen , Y. F. Zhang , and Z. R. Luo . 2025. “Effects of Simulated Nitrogen Deposition on the Competitive Relationship Between Invasive *Ageratum conyzoides* L. and Its Co‐Occurring Indigenous Forb *Acalypha australis* L.” Journal of Biosafety 34, no. 1: 53–59.

[ece371532-bib-0004] Chen, X. B. , Y. X. Zhang , Y. F. Zhang , and Z. R. Luo . 2023. “Response of Phenotypic Plasticity of Invasive *Ageratum conyzoides* L. to Interspecific Competition.” Plant Science Journal 41, no. 1: 37–43.

[ece371532-bib-0005] Davidson, A. M. , M. Jennions , and A. B. Nicotra . 2011. “Do Invasive Species Show Higher Phenotypic Plasticity Than Native Species and, If So, Is It Adaptive? A Meta‐Analysis.” Ecology Letters 14, no. 4: 419–431. 21314880 10.1111/j.1461-0248.2011.01596.x

[ece371532-bib-0006] Ferenc, V. , C. Merkert , F. Zilles , and C. S. Sheppard . 2021. “Native and Alien Species Suffer From Late Arrival, While Negative Effects of Multiple Alien Species on Natives Vary.” Oecologia 197, no. 1: 271–281. 34410489 10.1007/s00442-021-05017-3 PMC8445876

[ece371532-bib-0007] Gong, W. N. , F. H. Wan , B. Y. Xie , and J. Y. Guo . 2009. “Phenotypic Plasticity and Adaptability of the Invasive Alien Species.” Plant Protection 35, no. 4: 1–7.

[ece371532-bib-0008] Kaur, A. , S. Kaur , H. P. Singh , et al. 2023. “Ecology, Biology, Environmental Impacts, and Management of an Agro‐Environmental Weed *Ageratum conyzoides* .” Plants 12, no. 12: 2329–2343. 37375954 10.3390/plants12122329 PMC10301300

[ece371532-bib-0009] Keddy, P. , C. Gaudet , and L. H. Fraser . 2000. “Effects of Low and High Nutrients on the Competitive Hierarchy of 26 Shoreline Plants.” Journal of Ecology 88, no. 3: 413–423.

[ece371532-bib-0010] Khatri, K. , K. Bargali , B. Negi , A. Fartyal , and S. S. Bargali . 2024. “Evidences of Phenotypic Plasticity,and Lack of Local Adaptation and Clinal Differentiation in Invasive Ageratina Adenophora From Uttarakhand Himalaya.” Environment, Development and Sustainability. 10.1007/s10668-024-05733-9.

[ece371532-bib-0011] Khatri, K. , B. Negi , K. Bargali , and S. S. Bargali . 2023. “Phenotypic Variation in Morphology and Associated Functional Traits in *Ageratina adenophora* Along an Altitudinal Gradient in Kumaun Himalaya, India.” Biologia 78, no. 5: 1333–1347.

[ece371532-bib-0012] Li, H. R. , J. Yan , C. Du , and X. L. Yan . 2022. “Current Status and Suggestions of Research on Invasive Risk Assessment of Alien Plants in China.” Acta Ecologica Sinica 42, no. 16: 6451–6463.

[ece371532-bib-0014] Liu, M. Q. , X. F. Yang , Y. M. Shi , Y. W. Liu , X. M. Li , and W. J. Liao . 2022. “Effects of Simulated Acid Rain on the Competitive Relationship Between Invasive *Ambrosia artemisiifolia* and Its Co‐Occurring Indigenous Forb *Bidens bipinnata* .” Chinese Journal of Plant Ecology 46, no. 8: 932–940.

[ece371532-bib-0015] Luo, Z. R. , X. R. Chen , G. S. Xia , and X. B. Chen . 2018. “Extrinsic Environmental Factors, Not Resident Diversity Itself, Lead to Invasion of *Ageratum conyzoides* L. in Diverse Communities.” Ecological Research 33, no. 6: 1245–1253.

[ece371532-bib-0016] Lv, Y. , G. Q. Wang , L. Zheng , and H. W. Ni . 2011. “Competitiveness of Invasive Plant *Flaveria bidentis* With Native Weed Plants.” Chinese Journal of Ecology 30, no. 4: 677–681.

[ece371532-bib-0017] Negi, B. , S. S. Bargali , K. Bargali , and K. Khatri . 2020. “Allelopathic Interference of *Ageratum conyzoides* L. Against Rice Varieties.” Current Agriculture Research Journal 8, no. 2: 69–76.

[ece371532-bib-0018] Negi, B. , K. Khatri , S. S. Bargali , and K. Bargali . 2024. “Factors Determining the Invasion Pattern of *Ageratina adenophora* Spreng. In Kumaun Himalaya India.” Environmental and Experimental Botany 228: 106027.

[ece371532-bib-0019] Negi, B. , K. Khatri , S. S. Bargali , K. Bargali , A. Fartyal , and R. K. Chaturvedi . 2023. “Impact of Invasive *Ageratina adenophora* on Relative Performance of Woody Vegetation in Different Forest Ecosystems of Kumaun Himalaya, India.” Journal of Mountain Science 20, no. 9: 2557–2579.

[ece371532-bib-0044] Negi, B. , K. Khatri , S. S. Bargali , K. Bargali , and A. Fartyal . 2025. “Phenological Behaviour of *Ageratina adenophora* Compared With Native Herb Species Across Varied Habitats in the Kumaun Himalaya.” Plant Ecology 226, no. 3: 237–249.

[ece371532-bib-0021] Pan, Y. M. , S. C. Tang , C. Q. Wei , X. Q. Li , and S. H. Lv . 2022. “Competition Between Three Native Plants and Invasive *Ageratina adenophora* .” Acta Ecologica Sinica 42, no. 6: 2394–2404.

[ece371532-bib-0022] Puy, J. , F. de Bello , H. Dvorakova , N. G. Medina , V. Latzel , and C. P. Carmona . 2021. “Competition‐Induced Transgenerational Plasticity Influences Competitive Interactions and Leaf Decomposition of Offspring.” New Phytologist 6: 3497–3507. 10.1111/nph.17037 33111354

[ece371532-bib-0023] Rathee, S. , M. Ahmad , P. Sharma , et al. 2021. “Biomass Allocation and Phenotypic Plasticity Are Key Elements of Successful Invasion of *Parthenium hysterophorus* at High Elevation.” Environmental and Experimental Botany 184: 104392.

[ece371532-bib-0024] Schwinning, S. , and J. Weiner . 1998. “Mechanisms Determining the Degree of Size Asymmetry in Competition Among Plants.” Oecologia 113, no. 4: 447–455. 28308024 10.1007/s004420050397

[ece371532-bib-0025] Skácelová, O. , and J. Lepš . 2014. “The Relationship of Diversity and Biomass in Phytoplankton Communities Weakens When Accounting for Species Proportions.” Hydrobiologia 724, no. 1: 67–77.

[ece371532-bib-0026] Valladares, F. , C. C. Bastias , O. Godoy , E. Granda , and A. Escudero . 2015. “Species Coexistence in a Changing World.” Frontiers in Plant Science 6: 866. 26528323 10.3389/fpls.2015.00866 PMC4604266

[ece371532-bib-0027] Valladares, F. , D. Sanchez‐gomez , and M. Zavala . 2006. “Quantitative Estimation of Phenotypic Plasticity: Bridging the Gap Between the Evolutionary Concept and Its Ecological Applications.” Journal of Ecology 94, no. 6: 1103–1116.

[ece371532-bib-0028] Van Kleunen, M. , E. Weber , and M. Fischer . 2010. “A Meta‐Analysis of Trait Differences Between Invasive and Non‐Invasive Plant Species.” Ecology Letters 13, no. 2: 235–245. 20002494 10.1111/j.1461-0248.2009.01418.x

[ece371532-bib-0029] Wang, J. P. , L. J. Dong , and W. G. Sang . 2012. “Effects of Different Nitrogen Regimes on Competition Between *Ambrosia artemisiifolia* , an Invasive Species, and Two Native Species, *Artemisia annua* and *Artemisia Mongolica* .” Biodiversity Science 20, no. 1: 3–11.

[ece371532-bib-0030] Wang, K. F. 2021. “Competitive Effects Between Three Plant Species and Invasive Plant *Cenchrus pauciflorus* .” Journal of Hubei Minzu University (Natural Science Edition) 39, no. 2: 134–139.

[ece371532-bib-0031] Wang, S. , and D. W. Zhou . 2017. “Research on Phenotypic in Plants: An Overview of History, Current Status, and Development Trends.” Acta Ecologica Sinica 37, no. 24: 8161–8169.

[ece371532-bib-0032] Wang, Y. , W. H. Li , D. Li , and Z. Zhang . 2015. “Research Progresson Invasion Mechanism and Prevention Strategy of *Alternanthera philoxeroides* .” Journal of Zhejiang A&F University 32, no. 4: 625–634.

[ece371532-bib-0033] Weigelt, A. , and P. Jolliffe . 2003. “Indices of Plant Competition.” Journal of Ecology 91, no. 5: 707–720.

[ece371532-bib-0034] Xiong, Y. Q. , and C. Y. Zhao . 2022. “Phenotypic Plasticity and the Successful Invasion of Alien Plants.” Chinese Journal of Ecology 39, no. 11: 3853–3864.

[ece371532-bib-0035] Xu, H. X. , and M. Ma . 2021. “Comparison of Interspecific Competitive Ability Between *Xanthium italicum* Moretti and *Glycyrrhiza Uralensis* Fisch.” Acta Ecologica Sinica 41, no. 16: 6644–6653.

[ece371532-bib-0036] Xu, W. N. , S. S. Zheng , Q. J. Yu , and Z. R. Luo . 2019. “The Effects of Environmental Factors on Morphology, Survival and Fecundity of *Ageratum conyzoides* .” Journal of Lishui University 41, no. 5: 34–40.

[ece371532-bib-0042] Yang, X. , H. S. Zhou , Y. N. Liang , et al. 2024. “Effects of Nitrogen Addition on the Growth and Competition of Invasive Plant *Bidens pilosa* With Different Invasion Proportions.” Pratacultural Science 23, no. 1: 267–282. 10.11829/j.issn.1001-0629.2024-0266.

[ece371532-bib-0037] Yu, L. D. , Z. C. Zhu , and X. Y. Pan . 2020. “Phenotypic Plasticity of *Alternanthera philoxeroides* in Response to Root Neighbors of Kin: Introduced vs. Native Genotypes.” Biodiversity Science 28, no. 6: 651–657.

[ece371532-bib-0038] Zhang, B. C. , Y. Peng , L. F. Zang , K. N. Qin , X. B. Li , and C. L. Sui . 2017. “Plasticity of *Alternanthera philoxeroides* in Response to Three Karst Habitats.” Guihaia 37, no. 6: 702–706.

[ece371532-bib-0039] Zhang, L. J. , and A. R. Lou . 2022. “Phenotypic Variation in Floral Traits of an Invasive Plant ( *Solanum rostratum* ) and Its Impact on Reproductive Fitness.” Scientia Sinica Vitae 52, no. 8: 1281–1291.

[ece371532-bib-0040] Zhang, Y. F. , Z. H. Zheng , X. B. Chen , and Z. R. Luo . 2022. “Niche Characteristics of the Invasive Pant *Ageratum conyzoides* and Its Commonly Associated Weeds.” Acta Ecologica Sinica 42, no. 9: 3727–3737.

[ece371532-bib-0041] Zhao, C. Y. , X. J. Zhao , X. Y. Liu , and J. S. Li . 2019. “Relationships Between Invasive Alien Grasses and Native Plants Across Different National Nature Reserves, Yunnan Province.” Journal of Plant Protection 46, no. 1: 122–129.

